# Symptoms of ADHD and Other Common Mental Disorders Influence Academic Success in South African Undergraduates

**DOI:** 10.1177/10870547241310659

**Published:** 2025-01-17

**Authors:** Nawal Mohamad, Kim-Louise Rousseau, Fatimah Dowlut, Milton Gering, Kevin G. F. Thomas

**Affiliations:** 1 University of Cape Town, South Africa

**Keywords:** academic success, ADHD, alcohol use, anxiety, common mental disorders, comorbidity, depression, university students

## Abstract

**Objective:**

ADHD symptoms are highly prevalent among university students. These symptoms, particularly the inattentive cluster, predispose students to poorer academic performance and worse academic adjustment. Moreover, ADHD symptoms are often comorbid with other common mental disorders; this comorbidity also leads to poor outcomes. South African students often have fewer resources to successfully transition to university. Hence, our longitudinal study used data from a sample of South African first-year undergraduate students to investigate (a) associations between ADHD symptoms and academic performance/adjustment, (b) separate influences of the inattentive and hyperactivity-impulsivity clusters on academic performance/adjustment, and (c) the influence of the combination of ADHD and psychiatric comorbidities on academic performance/adjustment.

**Method:**

We collected data three times through the first semester of 2023. Predictors within our regression models included sociodemographic variables, psychological variables (self-reported symptoms of ADHD, depression, anxiety, and risky alcohol use), and high school academic performance. Outcomes were first-semester GPA and self-reported academic adjustment (magnitude of change across the semester and overall adjustment at the end of the semester).

**Results:**

Analyses showed that, unlike academic performance (*N* = 506), magnitude of change in academic adjustment (*N* = 180) was significantly predicted by ADHD symptoms and the combination of ADHD (*p* = .02), depression (*p* < .001), and anxiety symptoms (*p* = .01). Inattentive ADHD symptoms predicted both academic performance and magnitude of change in academic adjustment.

**Conclusions:**

Our findings suggest that the presence of ADHD symptoms (both with and without other common mental disorders) is associated with a smaller magnitude of academic adjustment, and that the presence of inattentive symptoms of ADHD is associated with both poorer academic performance and smaller magnitude of academic adjustment. These findings are significant in informing future interventions targeting the academic outcomes of first-year university students.

Although ADHD is diagnosed most commonly in children and adolescents, over the past decade increasing numbers of cases have been observed in adults (see, e.g., Faraone et al., 2021; Schoeman & Liebenberg, 2017). This increase may be attributed to a broadening of diagnostic criteria as well as better awareness among clinicians of how symptoms might arise and present differently in adults (J. L. Young & Goodman, 2016; Zalsman & Shilton, 2016). University students are a distinct subset of adults who may be at particular risk for an ADHD diagnosis, perhaps due to the demands of university either revealing previously masked symptoms or provoking a previously non-existent pattern of symptoms (Lefler et al., 2020; Song et al., 2021). Moreover, in university students, the three core symptoms of ADHD (inattentiveness, hyperactivity, and impulsivity; American Psychiatric Association, 2013) are independent and interactive predictors of academic success^1^ (Henning et al., 2022; L. Weyandt et al., 2013).

Even in light of those facts, most research investigating the influence of mental health on academic success focuses mainly on mood, anxiety, or substance use disorders (see, e.g., Arnekrans et al., 2018; Awadalla et al., 2020). This research typically examines the independent influence of a single disorder (see, e.g., Bitew & Birhan, 2021; Tibus & Ledesma, 2021). Even those studies that report on multiple disorders frequently neglect to explain how combined effects of comorbid disorders could intensify negative effects on academic performance (Alonso et al., 2018; Auerbach et al., 2019).

Hence, the current study investigated how symptoms of ADHD, combined with symptoms of common comorbid psychiatric disorders (mood, anxiety, and substance use disorders), might predict academic success.

## ADHD, Comorbid Psychiatric Disorders, and Academic Success in University Students

Existing estimates suggest that the prevalence of ADHD as a standalone diagnosis in university students is in the range of 3% to 8%, a figure that tends to be higher than that observed in older adults (Fayyad et al., 2017; Romo et al., 2018; Vos & Hartman, 2022). Although students entering university with pre-existing ADHD diagnoses may have adopted effective compensation strategies, challenges at the tertiary education level may undermine these strategies, leading to symptom manifestation (Canela et al., 2017). Consequently, many studies identify ADHD as impairing academic outcomes; for instance, it appears to predict overall college readiness, poorer grades, and greater difficulty in adjusting to university demands (see, e.g., Canu et al., 2021; Dou et al., 2022; Green & Rabiner, 2013). Two articles using the same longitudinal dataset reported that American students with ADHD (a) consistently had lower grade point averages (GPAs) than their peers, (b) showed slower trajectories of academic progress over time, and (c) faced persistent challenges in later years of study (DuPaul et al., 2018, 2021). ADHD also predicts other outcomes, including poor sleep quality and executive dysfunction (e.g., disinhibition, disorganized planning, and emotional dysregulation), that may affect academic performance indirectly (Bolden et al., 2019; Gray et al., 2016; L. Weyandt et al., 2013).

In samples of university students (and, indeed, in the general population), ADHD is often comorbid with other psychiatric disorders—most prominently depression, anxiety, and substance use (Anastopoulos et al., 2018; Busch et al., 2019; Choi et al., 2022; Mochrie et al., 2020; Seo et al., 2022). This comorbidity is frequently associated with poor academic outcomes (see, e.g., Prevatt et al., 2015). For instance, Riboldi et al. (2022) found, using data from a sample of Italian university students (N = 1,943), that students who presented with symptoms of both ADHD and depression had lower GPAs, spent fewer hours studying, and made slower progress in their academic programs than those with low symptom counts on measures of ADHD and depression. Similarly, Arria et al. (2013) found that the increasing misuse of alcohol and cannabis contributed significantly to poor academic outcomes (e.g., lower GPA and class attendance) in university students with self-reported ADHD (*N* = 86; see also Rooney et al., 2012).

Research examining the effects on academic performance of particular ADHD symptom clusters and comorbid psychiatric disorders has delivered mixed results. In a Canadian sample of 3,688 first-year undergraduate students, Henning et al. (2022) found that symptoms of inattention were stronger predictors of academic underachievement and university attrition than symptoms of hyperactivity and impulsivity. Similarly, L. Weyandt et al. (2013) found that the combination of high levels of impulsivity, inattention, depression, and anxiety contributed to lower levels of academic success in American undergraduate students (*n* = 24). In contrast, Daffner et al. (2022) found no contribution of inattention or depressive symptomology to cumulative GPA or to retention among first-year American students (*N* = 228). Additionally, Norvilitis et al. (2010) reported that hyperactivity was a non-significant predictor of academic adjustment (e.g., organizational skills and executive functions) after controlling for depressive symptoms.

## The Current Study

The presence of ADHD is associated with serious challenges for university students. First-year students, in particular, are confronted with the novel and numerous demands of university, such as time management, daily life tasks, and interpersonal relationships. When these demands are compounded by the presence of ADHD (and, frequently, by comorbid psychiatric disorders), the university experience may be exceptionally challenging for those students (Credé & Niehorster, 2012; Eddy et al., 2021).

Given the context of the literature reviewed above, we used a longitudinal study design to test the following hypotheses: (1) There will be an association between more severe ADHD symptoms, as measured at the beginning of the academic year, and (a) poorer academic performance in the first semester, (b) smaller magnitude of change in academic adjustment over the course of the semester, and (c) worse overall academic adjustment at the end of the semester; (2) ADHD symptoms of inattention will be better predictors of (a) academic performance in the first semester, (b) smaller magnitude of change in academic adjustment over the course of the semester, and (c) worse overall academic adjustment at the end of the semester than ADHD symptoms of hyperactivity-impulsivity; and (3) the combination of more severe ADHD symptoms with more severe symptoms of depression, anxiety, and risky alcohol use will, compared to ADHD symptoms alone, be a stronger predictor of (a) academic performance in the first semester, (b) smaller magnitude of change in academic adjustment over the course of the semester, and (c) worse overall academic adjustment at the end of the semester.

Our sample was drawn from the population of undergraduate students at a large public South African university. Published literature in this field largely neglects the experience of university students in the global south and in low- and middle-income countries (LMICs), tending to focus instead on North American and Western European samples. Many South African students will face challenges encountered less frequently by their global north counterparts—for example, they are more likely to be first-generation university students, thus giving them fewer resources to aid in the transition from high school and making them less prepared for the demands of undergraduate living and learning (Motsabi et al., 2020; Uleanya & Rugbeer, 2020). Although we do not expect to observe a different pattern of associations between symptoms of common mental disorders and academic success from that reported in studies emerging from the global north, we do anticipate that within a South African sample, there will be different factors and additional environmental complexities influencing the prevalence and manifestation of common mental disorders and thereby impacting on academic adjustment and academic performance. For instance, the prevalence of exposure to childhood trauma is higher among South African than North American students, and there is a relatively high proportion of first-generation students among the population of South African undergraduates (McGowan & Kagee, 2013; Padmanabhanunni, 2020). Hence, this cohort might have an existing vulnerability toward the early onset of mental disorders, and this vulnerability might be triggered by difficulty adjusting to university without the social support offered by family members with tertiary education experience. Furthermore, resource-constrained academic contexts are often characteristic of LMICs, which means that at-risk students are less likely to be identified and to be offered counseling services and other protective measures (Evans-Lacko & Thornicroft, 2019; Reinders et al., 2021).

## Method

### Participants

#### Recruitment

We used convenience sampling and a departmental student research participation program to recruit undergraduates registered for a 2023 first-semester introductory psychology course. At one of the first week’s lectures, we presented details of the study (most importantly, that data would be collected using an online survey during three separate waves, each separated from the next by several weeks) and invited participation.

#### Eligibility Criteria

Participants were required to be 2023 first-time university enrollees (because the study involved adjustment to university, it required participants to be unfamiliar with the tertiary education context), and to be aged between 18 and 24 years (first-year undergraduates are most likely to fit into this age group, and the prevalence of mood disorders is particularly high in this age range; Davey, 2021).

Individuals not meeting these criteria were excluded using skip logic during Time Point 1 recruitment (i.e., the online survey redirected them to a disqualification page).

#### Sample Size and Attrition Across the Data Collection Time Points

**Figure 1.**
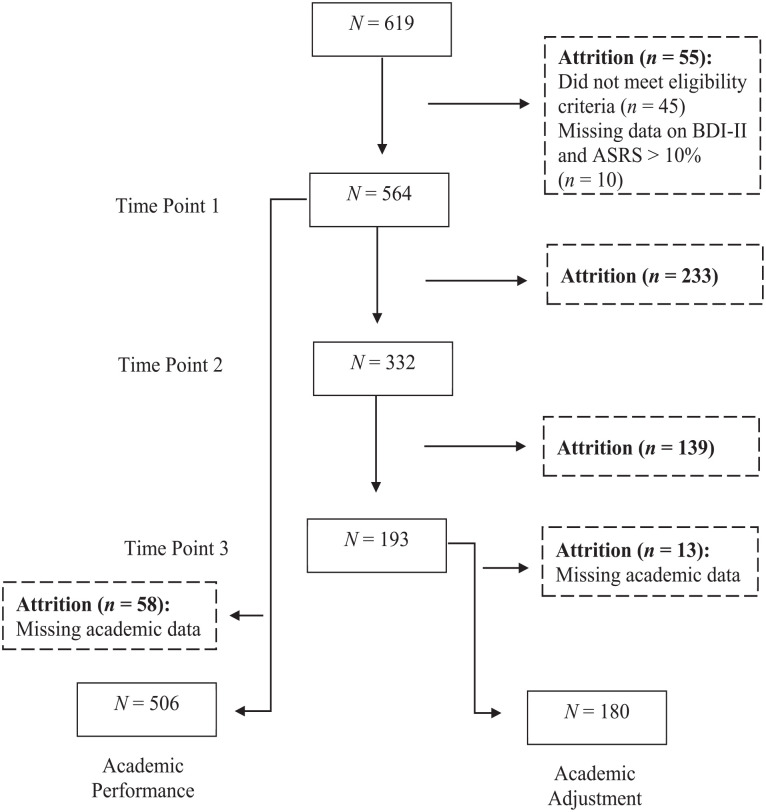
Participant recruitment flow and attrition across the data collection time points. *Note*. Participants excluded because of missing academic data were those for whom at least one of their National Benchmark Test scores, Grade 12 scores, and first-semester grades were unavailable or inaccessible. ASRS = Adult ADHD Self-Report Scale; BDI-II = Beck Depression Inventory-II.

The total sample size at the initial point of data collection was *N* = 619. However, due to a combination of factors (missing data, ineligible participants), we ended with a sample of 506 complete Time 1 datasets (see Figure 1). We used those data for analyses of the academic performance outcome variable. (Reasons and justification for this decision are given below.) Participation attrition from Time Point 1 to Time Point 2 to Time Point 3 meant we ended with a sample of 180 eligible participants who had completed the entire survey at all three measurement points and who had complete sets of academic data. We used data from those participants for analyses of the academic adjustment outcome variable. (Again, reasons and justification for this decision are given below.)

### Measures

#### Sociodemographic and Psychiatric History Questionnaire

This questionnaire gathered basic sociodemographic information (e.g., age, home language, sex, year of high school graduation, and first year of university) as well as information about history of psychiatric disorders, past and current psychiatric medication, and past and current psychotherapy.

#### Predictor Measures

##### High School Academic Performance

We accessed Grade 12 final examination scores and National Benchmark Test (NBT) results using the university’s student record database. The outcome variable for Grade 12 final examination scores was the average of the home language mark and the five highest other subject percentages (excluding Life Orientation), yielding a 0% to 100% score. The NBT is a standardized assessment, most often taken by students in the final year of high school, that is used by many South African universities as an admission criterion. The outcome variable here was the average of the percentages obtained for each of the *Quantitative Literacy*, *Academic Literacy*, and *Mathematics* subscales, yielding a 0% to 100% score.

##### Adult ADHD Self-Report Scale (ASRS)

This 18-item Likert-type scale (Kessler et al., 2005) assesses respondents’ ADHD symptoms and related feelings and behaviors over the 6 months prior to reporting. Items are based on current diagnostic criteria for ADHD in adults, with response options of *never* (assigned a score of 0), *rarely* (1), *sometimes* (2), *often* (3), and *very often* (4). Hence, total scores can range between 0 and 72; higher values indicate more severe symptoms. Items 1 to 4 and 7 to 11 assess inattention (Part A), while the other items assess hyperactivity-impulsivity (Part B). The developers reported a total classification accuracy of 97.9% (Kessler et al., 2005), and Hines et al. (2012) reported high sensitivity (1.00) and moderate positive predictive power (0.52). The ASRS has been used successfully in university student samples in Kenya and Iran (Atwoli et al., 2011; Shooshtari et al., 2021). It has good internal consistency reliability in South African clinical and non-clinical samples (α = .93 and .88, respectively; Regnart et al., 2019; Van Wijk, 2020).

##### Beck Depression Inventory-II (BDI-II)

This 21-item self-report instrument (Beck, 1996) assesses the presence of depressive symptoms in clinical and non-clinical populations. Each item evaluates the frequency and severity of a specific depressive symptom. Respondents choose, from a selection of four statements, the one that best describes their mental state over the previous 2 weeks. Each statement corresponds to a score: 0 = *no symptom presence*; 1 = *mild intensity* of symptoms; 2 = *moderate intensity* of symptoms; and 3 = *severe intensity* of symptoms. Hence, total scores can range from 0 to 63; higher values indicate more severe symptoms. In a meta-review, Wang and Gorenstein (2013) reported high internal consistency (α = .90) and test-retest reliability (*r* = .73–.96). These psychometric properties are retained in samples of South African university students (Makhubela & Debusho, 2016; Makhubela & Mashegoane, 2016; Rousseau et al., 2021).

##### Beck Anxiety Inventory (BAI)

This 21-item self-report instrument (Beck et al., 1988) assesses the presence and severity of anxiety symptoms. Each item evaluates a specific anxiety symptom. Respondents choose, from a selection of four statements, the one that best describes symptom persistence over the previous month. Each statement corresponds to a score: 0 = *not at all*; 1 = *mildly, but it didn’t bother me much*; 2 = *moderately*—*it wasn’t pleasant at times*; and 3 = *severely*—*it bothered me a lot*. Hence, total scores can range from 0 to 63; higher values indicate more severe symptoms. The BAI has strong internal consistency (α = .92) and good 1-week test-retest reliability (*r* = .75; Beck et al., 1988)). These properties are consistent in South African non-clinical samples (see, e.g., Kagee et al., 2015).

##### Alcohol Use Disorder Identification Test (AUDIT)

This 9-item self-report instrument (Babor et al., 2001; Saunders et al., 1993) assesses alcohol consumption, drinking behaviors, and alcohol-related problems. Respondents answer items about their drinking habits with their standard drinking behavior in mind. On seven items, respondents rate their drinking frequency (0 = *never*; 1 = *less than monthly*; 2 = *monthly*; 3 = *weekly*; and 4 = *daily or almost daily*). The other two items inquire whether anybody (including themselves) has been injured due to their drinking and whether somebody has indicated concern about their drinking habits. Hence, total scores can range from 0 to 40; higher values indicate a greater likelihood of harmful alcohol consumption. The AUDIT demonstrates good internal consistency (range for Cronbach’s α = .76–.96) in both clinical and non-clinical samples, including university students (Barry et al., 2015; López et al., 2019; Noorbakhsh et al., 2018). These psychometric properties are retained in South African university student samples (C. Young & Mayson, 2010).

#### Outcome Measures

##### Academic Adjustment Scale (AAS)

This 9-item self-report instrument (Anderson et al., 2016) measures confidence in academic adjustment to university demands. Respondents answer each item on a 5-point Likert-type scale, with anchors at 1 (*rarely applies to me*) and 5 (*always applies to me*), choosing the statement that best applies to them. The measure contains 3 subscales: (a) Academic Lifestyle (e.g., “*I am enjoying the lifestyle of being a student*”), (b) Academic Achievement (e.g., “*I am satisfied with my level of performance to date*”), and (c) Academic Motivation (e.g., “*I expect to successfully complete my degree in the usual allocated timeframe*”). Hence, total scores can range from 1 to 45; higher values indicate better academic adjustment. The developers report appropriate convergent, discriminant, and criterion validity, as well as good internal consistency and test-retest reliability (Anderson et al., 2016).

##### University Academic Performance

The variable, which was captured from the university’s student record database, was estimated using participants’ weighted GPA over their first semester courses. The weighting allowed us to consider the number of university credits allocated to each course (courses requiring more study hours [total hours include time taken to read, study, complete assignments, and take examinations] have more credits allocated to them). Previous research measuring the influence of psychiatric symptoms on university performance also used a cumulative grade as an outcome variable (see, e.g., Dou et al., 2022; DuPaul et al., 2021).

### Procedure

Ethical approval to conduct the study was granted by the relevant departmental and faculty authorities at our institution. All study protocols were conducted following the World Medical Association’s Declaration of Helsinki directives (World Medical Association, 2013). Participants were enrolled into the study only after they provided consent for (a) taking measures at Time Point 1, (b) being contacted for study activities at Time Point 2 and Time Point 3, and (c) accessing their high school and university academic records.

We collected data from participants at three separate time points within the first semester of 2023. The first wave of data collection (Time Point 1) ran between February 21 and March 7; the second (Time Point 2) began approximately 4 weeks later, running between April 4 and April 20; and the third (Time Point 3) began approximately 3.5 weeks after that, running between May 10 and May 24. Collection of data regarding high school and university academic performance measures was completed approximately 6 weeks after Time Point 3.

#### Time Point 1

Immediately after our in-class study presentation and participation invitation, we sent all students registered for the first-year psychology course a survey link. This survey, which was hosted on the SurveyMonkey platform (www.surveymonkey.com), comprised the appropriate consent forms and the standardized questionnaires listed above. Students who consented to participation were required to complete the survey in one sitting. Upon completion, they (a) were awarded a course credit, (b) received an interim debriefing form, and (c) were informed they would be contacted before the next wave of data collection. The survey took approximately 20 min to complete.

#### Time Point 2

Individuals who had completed the Time Point 1 measures were invited via email to complete another online survey. The Time Point 2 measures were identical to those administered at Time Point 1. This time, the opening and closing pages of the survey included an abbreviated consent form and an interim debriefing form, respectively. The latter document reminded participants they would be contacted before the next wave of data collection.

#### Time Point 3

Individuals who had completed both the Time Point 1 and Time Point 2 measures were invited via email to complete another online survey. The procedures here deviated from those for earlier time points only in terms of the debriefing form, which included final remarks and our contact details for any follow-up questions. This form also confirmed participants’ eligibility to receive financial compensation (ZAR50 for completing procedures through Time Point 2 and an additional ZAR50 for completing Time Point 3) for completing the study procedures.

### Statistical Preparation and Analyses

We used R Studio (R Core Team, 2022) to create the final data frames and to conduct all inferential statistical analyses. The threshold for statistical significance was set at α = .05. Where certain analyses comprised many significance tests, we interpreted statistically significant *p*-values greater than .001 with caution to minimize the increased risk of Type I errors.

#### Creating the Database and Deriving the Variables of Interest

To create a merged data frame, we combined the following: (a) sample sociodemographic information (age, home language, sex, final year of high school, and first year of university), (b) sample psychiatric information (history of psychiatric disorders, past and current psychiatric medication, and past and current psychotherapy), (c) other control variables (Grade 12 score and NBT score), (d) predictor variables (for each Time Point, ASRS total score as an estimate of ADHD symptoms; BDI-II total score as an estimate of depression; BAI total score as estimate of anxiety; and AUDIT total score as an estimate of risky alcohol use), and (e) outcome variables (GPA, AAS total score at each Time Point). We also added ASRS inattention (sum of items 1–4 and 7–11) and hyperactivity-impulsivity (sum of items 5–6 and 12–18) scores, following the proposed cluster organization of Kessler et al. (2005).

To derive one of the two primary academic adjustment outcome variables, we measured change over time rather than using each of the three AAS scores independently. To prepare this single change-over-time variable, we used mixed effects modeling. We chose this technique because of its suitability for repeated measures within individuals over time and its ability to account for within-subject variability. The AAS slope variable indicated non-significant change over time (*M* = 0, *SD* = 2.24, *p* > .05) and weak-to-moderate correlations over time (*r* = .38–.49, *p* < .001) with an intra-class correlation of .37, suggesting significant individual variability within the sample.

To test the main hypotheses, we conducted multiple regression models. This technique was suitable because the observations were cross-sectional and independent from each other.

#### Data Management

Regarding missing data, we found that in the Time Point 1 dataset 26 participants had not completed some ASRS items and 69 participants had not completed some BAI items. We excluded datasets with more than 10% of missing data in each of those scales (i.e., *n* = 11) and used the R mice (multivariate imputation by chained equations) package to impute the remaining observations. This technique replaces each missing value using a separate model (Van Buuren & Groothuis-Oudshoorn, 2011). For sensitivity purposes, we ran the analyses related to each a priori hypothesis with both the imputed data and missing observations, and both the weighted and unweighted GPA. The results were similar, and hence below we report only those based on datasets including imputed data points and the weighted GPA. There were no missing data in the Time Point 2 and the Time Point 3 datasets.

#### Descriptive Statistics and Other Preliminary Analyses

We created a full set of descriptive statistics that included measures of central tendency and variation for each variable contained in the merged data frame. These descriptives allowed us to gain an overall sense of the data with which we would be working and to confirm the suitability of the distributions for subsequent inferential analyses. We also conducted several between-group, correlational, and psychometric analyses whose results helped us confirm the appropriateness of our decision to use Time Point 1 data as the sole estimate of ADHD, depression, anxiety, and risky alcohol use symptoms. Results of those analyses are presented both below and in the Supplemental Material.

#### Primary Inferential Analyses

We used age, Grade 12 score, and NBT score as control variables in all statistical modeling. The major predictor variables (self-reported ADHD, depression, anxiety, and risky alcohol use symptoms) were, as noted earlier, based on Time Point 1 ASRS, BDI-II, BAI, and AUDIT total scores. As also noted earlier, weighted first semester GPA was the outcome variable estimating the construct of academic performance and the change in AAS score over the three time points was the outcome variable estimating the construct of academic adjustment.

##### Testing Hypothesis 1

Three separate linear regression models tested the hypothesis that there would be a significant association between more severe ADHD symptoms, as measured at the beginning of the year and (a) poorer academic performance in the first semester (i.e., lower grades in first-year university courses), (b) smaller magnitude of change in academic adjustment over the course of the semester, and (c) worse overall academic adjustment at the end of the semester. All three models featured ASRS total score at Time Point 1 as the key predictor, and age, Grade 12 score, and NBT score as control variables. The first model’s outcome variable was academic performance, as indexed by the weighted first semester GPA. The second model’s outcome variable was academic adjustment, as indexed by the AAS change-over-time variable. The third model’s outcome variable was academic adjustment, as indexed by the AAS score at Time Point 3. Where results were statistically significant, we assessed the effect sizes for the proportion of contribution to the outcome variable.

##### Testing Hypothesis 2

Six separate linear regression models tested the hypothesis that ADHD symptoms of inattention would be better predictors of academic performance (i.e., weighted first-semester GPA), smaller magnitude of academic adjustment than ADHD symptoms of hyperactivity-impulsivity, and overall academic adjustment at the end of the semester. The first three models featured ASRS Inattention subscale score at Time Point 1 as the key predictor, and age, Grade 12 score, and NBT score as control variables. The second three models differed from this only in that they featured ASRS Hyperactivity-Impulsivity subscale score at Time Point 1 as the key predictor. The first and fourth models’ outcome variable was academic performance, as indexed by weighted first-semester GPA. The second and fifth models’ outcome variable was academic adjustment, as indexed by the AAS change-over-time variable. The third and sixth models’ outcome variable was academic adjustment, as indexed by the AAS score at Time Point 3. Where results were significant, we assessed the effect sizes for the proportion of contribution to the outcome variable.

##### Testing Hypothesis 3

Three separate hierarchical regression models tested the hypothesis that the combination of ADHD symptoms with symptoms of depression, anxiety, and risky alcohol use would be a more significant predictor of (a) academic performance (i.e., weighted first-semester GPA), (b) change in academic adjustment, and (c) overall academic adjustment at the end of the semester than ADHD alone. For each outcome variable, the first block of the model contained ASRS total (Time Point 1), while the second contained ASRS, BDI-II, BAI, and AUDIT total scores (all Time Point 1). Where results were significant, we assessed the effect sizes and change in variance for the proportion of contribution to the outcome variable.

We considered running regression models that included ASRS subscale scores rather than total score. However, ASRS Inattention subscale scores were significantly positively associated with BDI-II and BAI total scores and hence the coefficient estimates of the models would be at risk of instability due to multicollinearity amongst predictor variables.

#### Power Analysis

An a priori analysis, using G*Power 3.1 software (Faul et al., 2009), featured the following parameters: linear multiple regression analysis, *R*^2^ deviation from zero, seven predictor variables (Grade 12 score, NBT score, age, ADHD, depression, anxiety, and risky alcohol use symptoms), and α = .05. The effect size, chosen based on findings from similar research (see, e.g., Daffner et al., 2022; DuPaul et al., 2018), was Cohen’s *f* = 0.15 (medium magnitude). The software calculated that *N* = 153 would be sufficient to achieve statistical power of 0.95. Hence, this study is adequately powered.

## Results

### Sample Sociodemographic Characteristics

As Table 1 shows, the average age of the sample at Time Point 1 (*N* = 506) was not significantly different from that at Time Point 2 (*N* = 332) and at Time Point 3 (*N* = 180). Similarly, the sex distribution was not substantially different from Time Point 1 to Time Point 2 to Time Point 3—most participants identified as female. These data patterns indicate there is little possibility that participant age or sex was predictive of the likelihood of dropping out of the study after Time Point 1. At Time Point 1, most participants reported English as their home language (*n* = 298, 58.89%), followed by isiXhosa (*n* = 77, 15.22%) and then isiZulu (*n* = 37, 7.31%) and Afrikaans (*n* = 28, 5.53%). The rest of the sample (*n* = 66, 13.04%) spoke other South African languages (e.g., Sepedi, Setswana, Sesotho).

**Table 1.** Descriptive Statistics: Sociodemographic Characteristics of the Sample at Each Data Collection Point.

Variable/statistic	Time point					
	1	2		3		
	(*N* = 506)	*f* (%)	(*N* *=* 332)	*f* (%)	(*N* = 180)	*f* (%)
Age						
* M*	18.43	—	18.44	—	18.47	—
* SD*	0.72	—	0.72	—	0.80	—
Range	18–22	—	18–22	—	18–22	—
Sex						
Female	412	81.42	287	86.44	160	88.89
Male	83	16.40	39	11.75	18	10.00
Non-binary	11	2.17	6	1.79	2	1.11

Analyses detected, for most outcome variables, no significant differences between those who completed the study (i.e., the Time Point 3 sample, *n* = 180) and those who did not (i.e., those who completed only the Time Point 1 and Time Point 2 measures, *n* = 326; see Table 2).

**Table 2.** Differences in the Main Outcome Variables Between Completers (*n* = and 180) Non-Completers (*n* = 326).

Outcome variable	Group	Mean	*SD*	95% CI	*p*
BDI-II	Non-completers^a^	16.33	10.45	[−1.69, 2.06]	.85
	Completers^b^	16.15	10.09		
BAI	Non-completers^a^	21.45	13.10	[−1.52, 3.12]	.50
	Completers^b^	20.65	12.06		
ASRS	Non-completers^a^	35.80	12.03	[−1.61, 2.59]	.65
	Completers^b^	35.31	10.50		
AUDIT	Non-completers^a^	3.96	5.19	[−.43, 1.32]	.32
	Completers^b^	3.52	4.08		
GPA^c^	Non-completers^a^	62.52	11.53	[−4.68, −0.41]	.02*
	Completers^b^	65.07	11.98		

*Note.* SD = standard deviation; CI = confidence interval; ASRS = Adult ADHD Self-Report Scale; BDI-II = Beck Depression Inventory-II; BAI = Beck Anxiety Inventory; AUDIT = Alcohol Use Disorder Identification Test; AAS = Academic Adjustment Scale.

a Values are for Time Point 1 and 2 data. Possible score ranges are: ASRS, 0–70; BDI-II, 0–58; BAI, 0–62; AUDIT, 0–28.

b Values are for Time Point 3 data. Possible score ranges are: ASRS, 0–70; BDI-II, 0–58; BAI, 0–62; AUDIT, 0–28.

c Variable derived from the weighted average grade across all first-semester courses.

* *p* < .05.

### Descriptive Statistics and Other Preliminary Analyses

Our analyses identified no concerning outliers. All distributions met the assumptions underlying the planned inferential statistical testing.

Measures of internal consistency indicated that, for each of the five main self-report measures, items were reliably measuring the same construct. For the AAS, Ω = .74; ASRS, Ω = .88; BDI-II, Ω = .90; BAI, Ω = .92; and AUDIT, Ω = .85.

Table 3 presents descriptive statistics for the study’s key control (except age, see Table 1), predictor, and outcome variables. Of particular interest here are data for the psychiatric variables. On average, the total BDI-II score was in the range (14–19 points) described as “mild depression” and the total BAI score was in the range (16–25 points) described as “moderate anxiety” (Beck, 1996; Maust et al., 2012). On average, the total AUDIT score was in the range (1–7 points) classifying individuals as having a low risk of hazardous alcohol consumption (Conigrave et al., 1995). The ASRS does not feature standardized cut-off scores for symptom severity.

**Table 3.** Descriptive Statistics: Scores on Control, Predictor, and Outcome Variables.

Variable Type / Name	Mean	Median	*SD*	Range	95% CI
Control					
Grade 12 score^a^	74.31	74	6.36	58–92	[73.76, 74.86]
NBT score^b^	58.66	57	11.30	33–88	[57.68, 59.64]
Predictor					
ASRS^c^	35.67	35	11.51	0–70	[34.67, 36.67]
BDI-II^c^	16.31	15	10.31	0–58	[15.41, 17.21]
BAI^c^	21.21	20	12.76	0–62	[20.10, 22.32]
AUDIT^c^	3.80	2	4.82	0–28	[3.38, 4.22]
Outcome					
AAS^d^	0	0.31	2.24	−6.08–5.00	[−0.18, 0.18]
GPA^e^	63.54	65.17	11.41	1.50–88.34	[62.55, 64.53]

*Note. SD* = standard deviation; CI = confidence interval; NBT = National Benchmark Tests; ASRS = Adult ADHD Self-Report Scale; BDI-II = Beck Depression Inventory-II; BAI = Beck Anxiety Inventory; AUDIT = Alcohol Use Disorder Identification Test; AAS = Academic Adjustment Scale.

a Variable derived from the average percentage across the home language mark and the five highest other subject percentages (excluding Life Orientation).

b Variable derived from the average percentage across the Quantitative Literacy, Academic Literacy, and Mathematics subscales.

c Values are for Time Point 1 data. Possible score ranges are: ASRS, 0–70; BDI-II, 0–58; BAI, 0–62; AUDIT, 0–28. .

d Variable derived from the change in AAS scores over the three time points.

e Variable derived from the weighted average grade across all first-semester courses.

Table 4 presents pairwise correlations between the major variables of interest. Of particular note here is that high school academic performance was not significantly associated with ASRS scores. This piece of data justified our decision to use Grade 12 score as a control variable in subsequent modeling.

**Table 4.** Pairwise Correlations Between Major Variables of Interest.

Variable	1	2	3	4	5	6	7	8	9	10	11
Control											
1. Grade 12 score^a^	—										
2. NBT score^b^	.51***	—									
3. Age	−.01	.08*	—								
Predictor											
4. ASRS^c^	−.04	.05	.05	—							
5. Inattention^c^	−.09*	−.04	−.05	.89***	—						
6. Hyperactivity-impulsivity^c^	.02	.14**	.02	.88***	.57***	—					
7. BDI-II^c^	−.14***	−.12***	−.03	.49***	.49***	.37***	—				
8. BAI^c^	−.05	−.10*	.02	.49***	.44***	.42***	.66***	—			
9. AUDIT^c^	.04	.21***	.03	.16***	.12**	.16***	.10*	.11**	—		
Outcome											
10. AAS^d^	.06	.11	.04	−.21***	−.32***	−.04	−.32***	−.08	.03	—	
11. AAS^e^	.13	.04	.00	−.11	−.16*	−.03	−.25***	−.02	−.04	.74***	—
12. GPA^f^	.51***	.26***	.11*	−.09	−.13**	−.02	−.20***	−.09	−.05	.24***	.24***

*Note.* NBT = National Benchmark Tests; ASRS = Adult ADHD Self-Report Scale; BDI-II = Beck Depression Inventory-II; BAI = Beck Anxiety Inventory; AUDIT = Alcohol Use Disorder Identification Test; AAS = Academic Adjustment Scale.

a Variable derived from the average percentage across the home language mark and the five highest other subject percentages (excluding Life Orientation).

b Variable derived from the average percentage across the Quantitative Literacy, Academic Literacy, and Mathematics subscales.

c Values are for Time Point 1 data.

d Variable derived from the change in AAS scores over the three time points.

e Variable derived from the end of semester AAS score.

f Variable derived from the weighted average grade across all first-semester courses.

* *p* < .05. **p < .01. ***p < .001.

**Figure 2.**
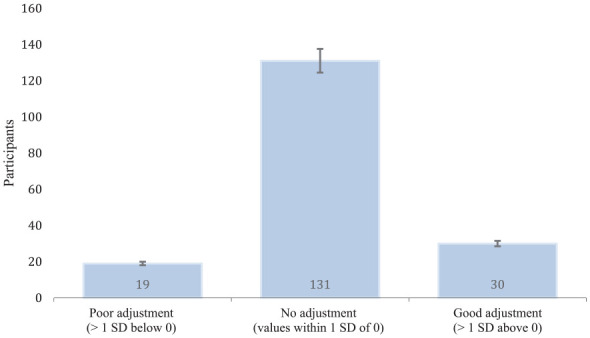
Self-reported academic adjustment between time point 3 and time point 1 (*N* = 180). *Note.* Scores were calculated by taking the difference in Academic Adjustment Scale (AAS) scores between Time Point 3 and Time Point 1 (*M* ± *SD* = 0 ± 5.53). The number for each data series represents the raw frequency of participants in that category. AAS = Academic Adjustment Scale.

Figure 2 presents the difference between the total AAS scores measured at Time Point 3 and Time Point 1. To assess the change in adjustment, we split the scores into three categories: <0 = *poor adjustment*, 0 = *no adjustment*, and >0 = *good adjustment*. This descriptive presentation of the frequency of the data was useful for interpreting the models and for identifying the trends in adjustment (i.e., those who showed worse adjustment, no adjustment, and improved adjustment) at the end of the semester.

### Testing Hypothesis 1

This hypothesis was partially supported. Although the analyses indicated that ADHD symptoms were not significant predictors of academic performance, *p* = .06, or of academic adjustment at the end of the semester, *p* = .13, they did detect a significant association between ASRS scores and magnitude of academic adjustment, *p* < .001. Specifically, higher symptom counts predicted a smaller magnitude of academic adjustment (see Table 5).

**Table 5.** Linear Regression Models: Association of ADHD Symptoms with Academic Performance (*N* = 506) and with Academic Adjustment (*N* = 180).

	*F*	Adj. *R*^2^	*t*	*p*	η_p_^2^
	Model 1: Academic performance				
Model fit	47.52	.27		<.001***	
Control variables					
Age			2.87	<.001***	.02
Grade 12 score^a^			11.40	<.001***	.26
NBT score^b^			0.02	.98	<.01
Predictor variable					
ASRS total score^c^			−1.87	.06	<.01
	Model 2: Change in academic adjustment ^d^				
Model fit	3.03	.04		< .01**	
Control variables					
Age			0.31	.76	<.01
Grade 12 score^a^			0.61	.54	.01
NBT score^b^			0.78	.44	<.01
Predictor variable					
ASRS total score^c^			−3.06	<.001***	.05
	Model 3: Overall academic adjustment ^e^				
Model fit^f^	1.64	.04		.17	
Control variables					
Age			0.01	.99	<.01
Grade 12 score^a^			0.10	.09	.02
NBT score^b^			0.00	.90	<.01
Predictor variable					
ASRS total score^c^			0.05	.13	.01

*Note.* η_p_^2^ = partial eta squared (effect size estimate); NBT = National Benchmark Tests; ASRS = Adult ADHD Self-Report Scale.

a Variable derived from the average percentage across the home language mark and the five highest other subject percentages (excluding Life Orientation).

b Variable derived from the average percentage across the Quantitative Literacy, Academic Literacy, and Mathematics subscales.

c Values are for Time Point 1 data.

d Variable derived from the change in AAS scores over the three time points.

e Variable derived from the end of semester AAS score.

f Model 3 was not statistically significant, suggesting the presence of the control variables in the model suppressed the independent main effect of hyperactivity-impulsivity symptoms on academic adjustment at the end of the semester.

** *p* < .01. ***p < .001.

### Testing Hypothesis 2

This hypothesis was confirmed (see Tables 6 and 7). Analyses indicated that inattention symptoms were better predictors of academic performance than hyperactivity-impulsivity symptoms (*p* = .04, η_p_^2^ < .01 versus *p* = .21, η_p_^2^ < .01). There was also a significant association between inattention scores and (a) magnitude of academic adjustment, *p* < .001, η_p_^2^ = .10 (specifically, higher symptom counts predicted smaller magnitude of academic adjustment), and (b) academic adjustment at the end of the semester, *p* = .04, η_p_^2^ = .02 (specifically, higher symptom counts predicted worse adjustment). In contrast, analyses detected no significant association between hyperactivity-impulsivity symptoms and (a) magnitude of academic adjustment, *p* = .29, η_p_^2^ < .01, or (b) academic adjustment at the end of the semester, *p* = .49, η_p_^2^ < .01.

**Table 6.** Linear Regression Models: Association of ADHD Inattention Symptoms with Academic Performance (*N* = 506) and with Academic Adjustment (*N* = 180).

	*F*	Adj. *R*^2^	*t*	*p*	η_p_^2^
	Model 1: Academic performance				
Model fit	47.75	.27		<.001***	
Control variables					
Age			2.81	.01 *	.02
Grade 12 score^a^			11.31	<.001***	.26
NBT score^b^			−0.10	.92	<.01
Predictor variable					
ASRS inattention subscale score^c^			−2.05	.04*	<.01
	Model 2: Change in academic adjustment^d^				
Model fit	5.67	.09		<.01**	
Control variables					
Age			0.06	.95	<.01
Grade 12 score^a^			0.12	.91	.01
NBT score^b^			0.48	.63	<.01
Predictor variable					
ASRS inattention subscale score ^c^			−4.45	<.001***	.10
	Model 3: Overall academic adjustment^e^				
Model fit^f^	2.08	.05		.09	
Control variables					
Age			−0.04	.93	<.01
Grade 12 score^a^			0.09	.15	.02
NBT score^b^			−0.01	.77	<.01
Predictor Variable					
ASRS Inattention subscale score^c^			−0.11	.04*	.02

*Note.* η_p_^2^ = partial eta squared (effect size estimate); NBT = National Benchmark Tests; ASRS = Adult ADHD Self-Report Scale.

a Variable derived from the average percentage across the home language mark and the five highest other subject percentages (excluding Life Orientation).

b Variable derived from the average percentage across the Quantitative Literacy, Academic Literacy, and Mathematics subscales.

c Values are for Time Point 1 data.

d Variable derived from the change in AAS scores over the three time points.

e Variable derived from the end of semester AAS score.

f Model 3 was not statistically significant, suggesting the presence of the control variables in the model suppressed the independent main effect of hyperactivity-impulsivity symptoms on academic adjustment at the end of the semester.

* *p* < .05. **p < .01. ***p < .001.

**Table 7.** Linear Regression Models: Association of ADHD Hyperactivity-Impulsivity Symptoms with Academic Performance (*N* = 506) and with Academic Adjustment (*N* = 180).

	*F*	Adj. *R*^2^	*t*	*p*	η_p_^2^
	Model 1: Academic performance				
Model fit	46.86	.27		<.001***	
Control variables					
Age			2.94	<.001***	.02
Grade 12 score^a^			11.50	<.001***	.26
NBT score^b^			0.03	.98	<.01
Predictor variable					
ASRS hyperactivity-impulsivity subscale score^c^			−1.26	.21	<.01
	Model 2: Change in academic adjustment^d^				
Model fit	0.93	<.001		.45	
Control variables					
Age			0.57	.57	<.01
Grade 12 score^a^			0.94	.35	.01
NBT Results^b^			0.63	.53	<.01
Predictor variable					
ASRS hyperactivity-impulsivity subscale score^c^			−1.05	.29	<.01
	Model 3: Overall academic adjustment^e^				
Model fit^f^	1.16	.03		.33	
Control variables					
Age			0.06	.89	<.02
Grade 12 score^a^			0.11	.06	.02
NBT results^b^			−0.01	.87	<.01
Predictor variable					
ASRS hyperactivity-impulsivity subscale score ^c^			−0.04	.49	<.01

*Note.* η_p_^2^ = partial eta squared (effect size estimate); NBT = National Benchmark Tests; ASRS = Adult ADHD Self-Report Scale.

a Variable derived from the average percentage across the home language mark and the five highest other subject percentages (excluding Life Orientation).

b Variable derived from the average percentage across the Quantitative Literacy, Academic Literacy, and Mathematics subscales.

c Values are for Time Point 1 data.

d Variable derived from the change in AAS scores over the three time points.

e Variable derived from the end of semester AAS score.

f Model 3 was not statistically significant, suggesting the presence of the control variables in the model suppressed the independent main effect of hyperactivity-impulsivity symptoms on academic adjustment at the end of the semester.

* *p* < .05. **p < .01. ***p < .001.

### Testing Hypothesis 3

This hypothesis was partially supported. The first hierarchical regression model indicated that neither overall ADHD symptoms alone nor overall ADHD symptoms in combination with symptoms of depression, anxiety, and risky alcohol use were significant predictors of academic performance (see Table 8). Although the model fit was statistically significant (*F*[7, 498] = 28.82, *p* < .001, *R*^2^ = .29), this observation is accounted for almost entirely by the influence of two control variables (Grade 12 score, age); the other predictor variables (with the exception of BDI-II score, which was significant on its own) accounted for no significant portion of the variance in the outcome, all *ps* > .10.

**Table 8.** Hierarchical Regression Model: Assessing Influence of ADHD and Comorbid Symptoms Versus ADHD Symptoms Alone in Predicting Academic Performance (*N* = 506).

Model	Unstandardized coefficients		Standardized coefficients	*t*	*p*
	*B*	*SE B*	β		
Block 1					
Control variables					
Age	1.74	0.61	.11	2.87	<.001***
Grade 12 score^a^	0.91	0.08	.51	11.40	<.001***
NBT score^b^	0.00	0.04	.00	0.02	.98
Predictor variables					
ASRS total score^c^	−0.07	0.04	−.07	−1.87	.06
Block 2					
Control variables					
Age	1.72	0.60	.11	2.85	<.001***
Grade 12 score^a^	0.87	0.08	.49	10.90	<.001***
NBT score^b^	0.01	0.05	.00	0.17	.87
Predictor variables					
ASRS total score^c^	−0.01	0.05	−.02	−0.33	.74
BDI-II total score^c^	−0.14	0.06	−.13	−2.39	.02*
BAI total score^c^	0.03	0.05	.03	0.57	.57
AUDIT total score^c^	−0.14	0.09	−.06	−1.55	.12
Model summary					
Block	*R* ^2^	Adj. *R*^2^	Change statistics		*p*
			Δ*R*^2^	Δ*F*	
1	.28	.27	.01	−14.36	<.001***
2	.29	.28	.01	−18.70	<.001***

*Note.* NBT = National Benchmark Test; ASRS = Adult ADHD Self-Report Scale; BDI-II = Beck Depression Inventory; BAI = Beck Anxiety Inventory; AUDIT = alcohol use disorder identification test.

a Variable derived from the average percentage across the home language mark and the five highest other subject percentages (excluding Life Orientation).

b Variable derived from the average percentage across the Quantitative Literacy, Academic Literacy, and Mathematics subscales.

c Values are for Time Point 1 data.

*** *p* < .001.

However, the second hierarchical analysis indicated that the combination of overall ADHD symptoms with symptoms of depression, anxiety, and risky alcohol use was a stronger predictor of a smaller magnitude of academic adjustment than overall ADHD symptoms alone (see Table 9). This time, the statistically significant model fit (*F*[7, 172] = 4.59, *p* ≤ .001, *R*^2^ = .16) was accounted for almost entirely by the contribution of ASRS, BDI-II, and BAI scores. Note that the AUDIT score was a non-significant predictor of this outcome.

**Table 9.** Hierarchical Regression Model: Assessing Influence of ADHD and Comorbid Symptoms Versus ADHD Symptoms Alone in Predicting Academic Adjustment (*N* = 180).

Model	Unstandardized coefficients		Standardized coefficients	*t*	*p*
	*B*	*SE B*	β		
Block 1					
Control variables					
Age	0.07	0.22	.02	.31	.76
Grade 12 score^a^	0.02	0.03	.05	.61	.54
NBT score^b^	0.01	0.02	.06	.78	.44
Predictor variables					
ASRS total score^c^	−0.05	0.02	−.22	−3.06	<.001***
Block 2					
Control variables					
Age	−0.03	0.21	−.01	−.14	.89
Grade 12 score^a^	0.00	0.03	.00	.04	.97
NBT score^b^	0.01	0.02	.07	.88	.38
Predictor variables					
ASRS total score^c^	−0.04	0.02	−.19	−2.28	.02*
BDI-II total score^c^	−0.09	0.02	−.39	−4.21	<.001***
BAI total score^c^	0.05	0.02	.25	2.63	.01**
AUDIT total score^c^	0.03	0.04	.05	.73	.47
Model summary					
Block	*R* ^2^	Adj. *R*^2^	Change statistics		*p*
			Δ*R*^2^	Δ*F*	
1	.06	.04	.05	2.15	<.05*
2	.16	.12	.10	1.56	<.001***

*Note.* NBT = National Benchmark Tests; ASRS = Adult ADHD Self-Report Scale; BDI-II = Beck Depression Inventory; BAI = Beck Anxiety Inventory; AUDIT = alcohol use disorder identification test.

a Variable derived from the average percentage across the home language mark and the five highest other subject percentages (excluding Life Orientation).

b Variable derived from the average percentage across the Quantitative Literacy, Academic Literacy, and Mathematics subscales.

c Values are for Time Point 1 data.

* *p* < .05. ***p < .001.

The third hierarchical regression model indicated that the combination of ADHD symptoms with symptoms of depression, anxiety, and risky alcohol use was a stronger predictor of academic adjustment at the end of the semester than ADHD symptoms alone (specifically, higher combined symptom counts predicted worse adjustment; see Table 10). This time, the statistically significant model fit (*F*[7, 172] = 3.39, *p* < .001, *R*^2^ = .12) was accounted for almost entirely by the contribution of BDI-II and BAI scores; ASRS and AUDIT scores were non-significant predictors of the outcome.

**Table 10.** Hierarchical Regression Model: Assessing Influence of ADHD and Comorbid Symptoms Versus ADHD Symptoms Alone in Predicting Academic Adjustment at Time Point 3 (*N* = 180).

Model	Unstandardized coefficients		Standardized coefficients	*t*	*p*
	*B*	*SE B*	β		
Block 1					
Control variables					
Age	0.01	0.41	.00	.02	.99
Grade 12 score^a^	0.10	0.06	.15	1.69	.09
NBT score^b^	0.00	0.03	−.01	−.13	.90
Predictor variables					
ASRS total score^c^	−0.05	0.03	−.12	−1.53	0.13
Block 2					
Control variables					
Age	−0.21	0.40	-.04	−.52	.61
Grade 12 score^a^	0.06	0.06	.09	1.07	.29
NBT score^b^	0.00	0.03	.01	.15	.88
Predictor variables					
ASRS total score^c^	−0.03	0.03	-.08	−.93	.36
BDI-II total score^c^	−0.16	0.04	-.38	−4.02	<.001***
BAI total score^c^	0.09	0.03	.26	2.70	.01**
AUDIT total score^c^	−0.01	0.08	-.01	−.14	.89
Model summary					
Block	*R* ^2^	Adj. *R*^2^	Change Statistics		*p*
			Δ*R*^2^	Δ*F*	
1	.04	.01	.02	.25	.17
2	.12	.09	.08	1.75	<.001***

*Note.* NBT = National Benchmark Tests; ASRS = Adult ADHD Self-Report Scale; BDI-II = Beck Depression Inventory; BAI = Beck Anxiety Inventory; AUDIT = alcohol use disorder identification test.

a Variable derived from the average percentage across the home language mark and the five highest other subject percentages (excluding Life Orientation).

b Variable derived from the average percentage across the Quantitative Literacy, Academic Literacy, and Mathematics subscales.

c Values are for Time Point 1 data.

** *p* < .01. ***p < .001.

## Discussion

The overarching aim of this research was to describe the influence of ADHD symptoms (with and without symptoms of comorbid psychiatric symptoms) on university students’ academic success. South African undergraduates (*N* = 506) completed self-report symptom measures of ADHD, depression, anxiety, and risky alcohol use, as well as an academic adjustment questionnaire, at three separate time points across the first semester of 2023. We used university databases to access information about the high school academic performance of those students, as well as their performance in first-semester university courses.

Using this design, we tested three hypotheses: (1) There will be an association between more severe ADHD symptoms, as measured at the beginning of the academic year, and (a) poorer academic performance in the first semester, (b) smaller magnitude of change in academic adjustment over the course of the semester, and (c) worse overall academic adjustment at the end of the semester; (2) ADHD symptoms of inattention will be better predictors of (a) academic performance in the first semester, (b) smaller magnitude of change in academic adjustment over the course of the semester, and (c) worse overall academic adjustment at the end of the semester than ADHD symptoms of hyperactivity-impulsivity; and (3) the combination of more severe ADHD symptoms with more severe symptoms of depression, anxiety, and risky alcohol use will, compared to ADHD symptoms alone, be a stronger predictor of (a) academic performance in the first semester, (b) smaller magnitude of change in academic adjustment over the course of the semester, and (c) worse overall academic adjustment at the end of the semester.

### ADHD and Academic Performance

Contrary to our a priori predictions, most analyses related to academic performance delivered results that were not statistically significant. Specifically, analyses suggested that (a) self-reported severity of ADHD symptoms was not significantly associated with academic performance (as measured by the weighted first-semester GPA), and (b) neither symptoms of ADHD alone nor the combination of ADHD and comorbid psychiatric symptoms (depression, anxiety, risky alcohol use) was a statistically significant predictor of academic performance. Broadly speaking, these results are inconsistent with previously published literature in this field.

Numerous previous studies indicate that university students with ADHD tend to experience difficulty achieving high levels of academic performance (see, e.g., Green & Rabiner, 2013; Lewandowski et al., 2021). For instance, Dou et al. (2022) found that more severe ADHD symptomatology (as measured by the ASRS) was a significant predictor of lower GPA in a sample of 617 Canadian undergraduates. There are several potential explanations for the discrepancy between our findings in this regard and those reported previously.

Most of these explanations center on methodological concerns (e.g., the design of the study, the nature of the sample, attrition). Regarding study design, outcome data collected over longer time periods may be more likely to show significant associations with ADHD symptomatology. For instance, students with ADHD symptoms may be more likely than those without to show consistently poor academic performance, especially in the more difficult courses that that they may take after the first year, and to drop out of university before completing the degree. This situation might arise because ADHD-diagnosed students tend to use academic assistance services (e.g., study skills workshops) less frequently and to take fewer classes in an attempt to manage their workload (DuPaul et al., 2018, 2021). Because our outcome measure of academic performance was indexed by a single variable that captured information about performance over a single semester, we may have been limited in the effects we could detect.

Regarding the nature of our sample, we did not recruit a group of participants with formal ADHD diagnoses and compare their academic performance and academic adjustment to those without such a diagnosis. Instead, we used a self-report measure to capture the continuum of ADHD symptom severity and used those data in regression models estimating their predictive power. In other words, where many other studies in this field used categorical data (i.e., ADHD versus non-ADHD) to explore their research questions (see, e.g., DuPaul et al., 2021; Lewandowski et al., 2013), we used continuous data (which by their nature dictated that the effects of subclinical ADHD would be included in effect estimates) to explore ours. Categorical data may permit group differences to be magnified (i.e., for the effects of formally diagnosed ADHD on academic performance to be properly distinguished; see, e.g., DuPaul et al., 2018; Lewandowski et al., 2021). Continuous data, on the other hand, permit investigation of the effects of subclinical as well as clinically diagnosed ADHD.

Additionally, we found that the academic performance of study completers (i.e., those who completed the measures at all three time points) was significantly better than that of study non-completers (i.e., those who did not complete measures at either Time Point 2 or Time Point 3). One interpretation of this finding is that those who completed the study were characterized by a higher general level of motivation, and that they therefore evinced a stronger desire to complete the study and to deliver a higher level of academic performance. Although there was considerable sample attrition (506 participants were enrolled and 180 [36%] completed the study), a mitigating factor is that we used Time Point 1 data for all modeling of the academic performance outcome variable. Moreover, our analyses indicate that there were no broad-based or systemic differences between study completers and study non-completers. That is to say, there were no significant between-group differences with regard to age and psychiatric symptoms (ADHD, depression, anxiety, and risky alcohol use) at baseline—only academic performance differed. Hence, it is possible (although we regard it as unlikely) that selective attrition by GPA scores could have contributed to the pattern of results.

A minor but noteworthy finding in our analyses was that there was a non-significant association between ADHD symptoms and high school academic performance. This finding is not consistent with those reported by numerous previous studies which together suggest that, in high school samples, an ADHD diagnosis (or the presence of significant ADHD symptoms) predicts relatively low levels of academic performance (see, e.g., Arnold et al., 2020; Fried et al., 2016; Zendarski et al., 2017). The reason for the difference between those findings and ours is likely due to sampling bias: Recall that we recruited our sample only after they had already gained entry into university; given that universities have strict admission criteria, those entering the institution are likely to have performed well in high school (Van der Westhuizen & Barlow-Jones, 2015; Van Zyl et al., 2012). Our sample had an average Grade 12 score of 74.31%, which might indicate that even if they experienced significant ADHD symptoms during high school, they might have employed effective compensation strategies. These strategies may remain effective when students with pre-existing ADHD enter university, leading to little symptom manifestation and a stable transition from high school (leading to satisfactory levels of academic performance [average first-semester GPA = 63.54%]) in this academically strong group.

The finding that the combination of symptoms of ADHD and other common mental disorders was a non-significant predictor of academic performance is inconsistent with previous research on university students (see, e.g., Anastopoulos et al., 2018; Prevatt et al., 2015; Riboldi et al., 2022). As noted above, it is likely that many of our participants did not meet the formal diagnostic criteria for ADHD and that subclinical symptomatology may not have the same disruptive effects as clinical symptoms. Moreover, our sample’s average scores on the BDI-II and BAI categorize them with only mild and moderate symptoms, respectively (Beck, 1996; Maust et al., 2012). One explanation for this pattern of data is that South African children and adolescents have relatively high levels of exposure to stressful and traumatic events, and that such exposure may elevate their risk for experiencing common mental disorders in university (McGowan & Kagee, 2013; Padmanabhanunni & Wiid, 2022). However, even on a global level, university students are likely to show an increase in symptoms of common mental disorders (see, e.g., Auerbach et al., 2016; Davey, 2021; Pedrelli et al., 2015). For instance, prior research on Cameroonian medical students (*N* = 618) presenting with mild depression symptoms reported no association with academic performance (Ngasa et al., 2017). Similarly, Muniz et al. (2022) found that lower levels of anxiety were not associated with poor academic performance in a Brazilian sample of undergraduate students (*N* = 331). Hence, the non-significant effects of combining generally mild and subclinical symptoms suggest that university students may have to present with symptoms that exceed clinical thresholds in order for academic performance to be affected negatively.

When ASRS total scores were separated into the conventional clusters, ADHD symptoms of inattention (but not ADHD symptoms of hyperactivity-impulsivity) were statistically significant predictors of academic performance. This finding is consistent with previous research on university students (see, e.g., Henning et al., 2022; Prevatt et al., 2015; L. Weyandt et al., 2013). We note, however, that some previous studies find no association between symptoms of inattention and academic success (see, e.g., Daffner et al., 2022).

Of interest here is research suggesting that sluggish cognitive tempo (SCT) eliminates or substantially reduces the association between inattention and academic performance (see, e.g., Becker & Barkley, 2018; Becker et al., 2014; Flannery et al., 2017). Although SCT is often comorbid with ADHD symptoms of inattention and with depression and anxiety, it is distinct from ADHD and is defined by symptoms of daydreaming, mental confusion, and slowed thinking (Becker & Barkley, 2018). Studies of North American university students indicate that SCT is associated with poorer learning and study skills, and that it accounts for substantial variance in executive dysfunction and functional impairment after controlling for depression, anxiety, and ADHD (Wood et al., 2017). Our study did not measure SCT; future studies may benefit from investigating relationships between that construct, inattention, and undergraduate academic performance.

Regarding hyperactivity-impulsivity, the analyses detected no significant association with both outcomes. This finding may be at least partially explained by the commonly observed decline in hyperactivity-impulsivity in adulthood (Prevatt et al., 2015). In our sample, there were lower average scores for hyperactivity-impulsivity (*M* = 15.20) than inattention (*M* = 20.32; total possible score range for both symptom clusters = 0–36).

### ADHD and Academic Adjustment

Our primary positive findings all related to the academic adjustment outcome variable (i.e., change in Academic Adjustment Scale [AAS; Anderson et al., 2016] total score across the three waves of data collection). Specifically, our analyses confirmed a priori predictions in suggesting that (a) greater self-reported severity of ADHD symptoms (as measured by the Adult ADHD Self-Report Scale [ASRS; Kessler et al., 2005] total score) was significantly associated with smaller magnitude of academic adjustment, (b) ADHD symptoms of inattention were stronger predictors of smaller magnitude of academic adjustment than ADHD symptoms of hyperactivity-impulsivity (more severe symptoms of inattention were significantly associated with smaller magnitude of academic adjustment, whereas there was no significant relationship between severity of hyperactive-impulsive symptoms and academic adjustment), and (c) the combination of ADHD and comorbid psychiatric symptoms was a stronger predictor of a smaller magnitude of academic adjustment than ADHD alone (specifically, when symptoms of depression {as measured by total score on the Beck Depression Inventory-II [BDI-II]; Beck, 1996} and anxiety {as measured by total score on the Beck Anxiety Inventory [BAI]; Beck et al., 1988} were present alongside symptoms of ADHD, more severe symptomatology was more strongly associated with a smaller magnitude of academic adjustment than when only ADHD symptoms were present). Broadly speaking, these results are all consistent with previously published literature in this field.

First, numerous previous studies indicate that university students with ADHD tend to experience difficulty adjusting to their academic environments (see, e.g., de Oliveira et al., 2016; Hazan-Liran & Miller, 2022). For instance, in a Brazilian sample of first-year university students (*N* = 18), the presence of ADHD symptoms appeared to make it more challenging to attend to academic tasks and demands (de Oliveira & Dias, 2017). Mechanisms that might contribute to this relationship are the relative inability to maintain attention, inhibit behavior, exercise self-control, and manage time effectively. Additionally, the increased academic demands of tertiary education over secondary education (e.g., managing schedules independently, coping with cyclical assessments, applying newly-acquired skills to novel situations) may heighten the demands for greater planning and organizational skills, making it more challenging to adjust to university (see, e.g., Dvorsky & Langberg, 2019; Fleming & McMahon, 2012). Hence, students experiencing these consequences of their ADHD symptoms might tend to have negative views of the academic experience and to adjust to it more poorly.

This poor academic adjustment outcome in students with ADHD symptoms could be exacerbated by the absence of positive psychological resources (e.g., high levels of intrinsic motivation, self-esteem, and university readiness), thus making the subjective interpretation of academic demands less optimistic (Ben-Naim et al., 2019; Hazan-Liran & Miller, 2022). To elaborate: A potential mechanism for impairment in these cases might be that students struggle with academic adjustment because they cannot allocate sufficient motivation to plan and realize their goals toward meeting academic requirements. Struggling to meet those requirements may lead to a negative feedback loop that ultimately creates a pessimistic outlook on the academic experience.

Second, several previous studies single out the inattentive presentation of ADHD as having a particularly negative effect on adjustment to the university academic environment and its requirements (see, e.g., L. L. Weyandt et al., 2017). For instance, Salla et al. (2019) found that ADHD-related inattention contributed to higher perceived stress and heightened academic concerns in a sample of French university students (*N* = 6,951). Similarly, a qualitative study of American undergraduate students (*n* = 12) reported that the inattentive presentation of ADHD was an important factor leading them to drop out of mainstream universities and to instead join a specialized training college for students with ADHD and learning disorders (Stamp et al., 2014). Although this was a small-sample study and did not compare the experiences of students with the inattentive presentation to those with the hyperactive-impulsive presentation, its findings provide valuable insight into self-reported reasons for perceived difficulties in academic adjustment. For example, students reported awareness of their differences from their peers, feelings of lack of understanding, and overall shame and anxiety for not displaying “normal” social behavior. Again, a negative feedback loop may be created: Insight into how symptoms of inattention might be affecting the social and academic experience might lead students to have poorer perceptions of their university readiness, which might lead to higher stress levels and consequent exacerbation of psychiatric symptoms.

Third, the finding that combined ADHD and depression/anxiety symptoms was a stronger predictor of academic adjustment than ADHD alone is consistent with existing research on university students indicating that the comorbidity of mood disorders leads to poorer adjustment to their student roles and poorer perceptions of their academic abilities (L. Weyandt et al., 2013). However, of interest here is that our analysis showed that the addition of comorbid psychiatric symptoms to the model increased its predictive power (as measured by the adjusted *R*^2^) from 6% to just 16%. In other words, the severity of common mental symptoms (ADHD, depression, anxiety) at the start of the first semester of university, although statistically significant and of clinical importance, does not account for most of the variance in academic adjustment of first-year university students across the semester.

We found, unexpectedly, a positive relationship between anxiety symptoms and changes in academic adjustment across the semester. Usually, higher levels of anxiety are associated with worse academic adjustment and lower levels of academic performance (Begdache et al., 2019; Larose et al., 2019). In the current case, however, we speculate that the motivation to succeed at university (as indexed by the high scores on the AAS Academic Motivation and Achievement subscales) might have tempered the negative effects of anxiety symptoms—in other words, higher levels of motivation might have inspired greater efforts to adjust to university and in so doing may have counteracted the impairing effects of anxiety (Balogun et al., 2017; Guo & Qiu, 2024). Naturally, this account is speculative; the association needs to be explored in different samples of first-year university students.

Additionally, our analyses suggest that ASRS scores (especially when considered in tandem with the control variables of age and high school academic performance) are non-significant predictors of academic adjustment as measured at the end of the semester. We speculate that participants may have considered how well they performed academically when self-reporting academic adjustment at Time Point 3. Recall that study completers had, on average, significantly higher GPAs than non-completers. Hence, against this background, perceptions of adjustment may be inflated, masking the effects of ADHD symptoms. These symptoms may instead have contributed to the overall trajectory of adjustment over the course of the semester (as measured by the change variable we employed in the main analyses).

### Limitations

First, psychometric analyses indicate that the AAS and AUDIT may not have been sufficiently sensitive measures of our variables of interest (see Supplemental Material). The AAS contained three items with poor inter-item correlations; removing these items led to the instrument becoming unidimensional, rendering the subscales redundant. The AUDIT may not be sensitive in identifying risky drinking patterns among first-year students because emerging adults could take more than one semester to establish such habits (Härkönen & Mäkelä, 2022). Although we tested the hypotheses using the first question of the AUDIT (measures drinking frequency) as a predictor of the outcomes, the findings were non-significant, indicating a need for a more suitable measure of alcohol use.

Second, participants were not recruited based on clinical diagnoses but rather on their self-reports. Broadly speaking, self-report scales tend to produce inaccurate behavioral data (Kormos & Gifford, 2014); of particular relevance with regard to university students is that they frequently misestimate the severity of their current experiences when reporting symptoms on forced-choice questionnaires (Althubaiti, 2016; Rousseau et al., 2021). Of further potential importance here is that our sample was predominantly female and that ADHD symptom reports sometimes show gendered patterns (see, e.g., Jaconis et al., 2016). Additionally, we did not gather data on whether participants were being treated for any impairing symptoms or whether they were taking performance-enhancing medication.

Third, the complete reliance on self-report measures to estimate all predictor variables and one major outcome variable (academic adjustment) increased the risk of shared-method variance and consequent inflation of the observed correlations (Spector et al., 2019). Of note, however, is that we took objective measures of academic performance and hence mitigated this risk to some degree.

Fourth, several unmeasured contextual factors (including social support, academic support, help-seeking behavior, and medication use) might have contributed to the observed data and influenced the results of our analyses. Social support (i.e., the level of support students receive from their friends, classmates, teachers, family, and other important people; de la Iglesia et al., 2014) is a strong predictor of both academic performance and adjustment in first-year university students (see, e.g., Berbery & O’Brien, 2018; Rodríguez et al., 2017; Silva et al., 2021). There is also a significant association between the provision of continuous academic support/coaching and academic performance. Students with ADHD who receive such support/coaching benefit not only in terms of their academic performance, but also in terms of their organizational skills and overall well-being (see, e.g., DuPaul et al., 2021; Field et al., 2013; Richman et al., 2014). Help-seeking behaviors (e.g., using tutoring services and other supplementary resources to improve studying methods and organizational skills) can also serve as protective factors guarding against poor academic outcomes. Motivation to succeed may be a protective factor that masks the influence of ADHD symptoms on academic performance (Prevatt et al., 2017; Prevatt & Yelland, 2015; Simon-Dack et al., 2016). Pharmacological treatments, such as Adderall or Ritalin, have been shown to improve cognitive outcomes in individuals with ADHD, improving vigilance, concentration, and inhibition (see, e.g., Pakdaman et al., 2018; L. L. Weyandt et al., 2014, 2017). Hence, the independent and interactive influence of such contextual factors is unknown in our sample.

Fifth, we did not collect data on the participants’ race and ethnicity. Our institution’s research ethics committee prohibits the collection of such data unless there is strong justification. Black and ethnic minority students are at higher risk of poor academic performance and of dropping out of university, largely due to historical socioeconomic inequities and ongoing systemic disparities (Bantjes et al., 2022, 2023; Murphy & Wyness, 2020). Hence, the independent and interactive influence of race and ethnicity is unknown in our sample.

Sixth, because more than 80% of our sample at all measurement time points self-identified as female and because we did not have enough statistical power to run independent analyses on students who self-identified otherwise, our findings may not be generalizable to students who self-identify as either male or non-binary. Hence, this sampling bias (which is a consequence of our recruitment pool [undergraduate psychology classes at our university] being comprised mostly of female students; Rousseau et al., 2021) limits our understanding of how sex could influence psychopathology, and of how that interaction might influence academic and other functional outcomes (Hartung & Lefler, 2019; Howard et al., 2017). Future research in this area will benefit by collecting more data from men and from sexual and gender minority populations.

Future research may also benefit from broadening the sampling frame to include senior undergraduate students. Second-, third-, and fourth-year undergraduate courses may be more rigorous, with escalated expectations in terms of both engagement and assessment, and hence academic performance in those courses may be more vulnerable to the negative effects of poor mental health. One consideration here, however, is that senior undergraduate samples may be biased as they will inevitably consist of those students who did not drop out during or immediately after the first year of study (the risk of drop-out at those stages is particularly high in South African universities; Alban & Mauricio, 2019; Mall et al., 2018; University of Cape Town, 2022) .

## Summary and Conclusion

This research contributes to scientific literature exploring the influence of mental health challenges on academic outcomes. We analyzed the influence of ADHD symptoms (with and without comorbid psychiatric disorders) on the academic success of first-year students at a South African university. We found that ADHD symptoms alone, as well as the combination of symptoms of ADHD and other common mental disorders, were strong predictors of academic adjustment but not academic performance. This does not mean to say that these symptoms of psychological distress will not influence academic performance in the long term. Our period of follow-up was relatively short (all measures were taken within a single semester), and hence the current design does not allow speculation about whether relatively poor adjustment during the first semester of university, combined with ongoing symptoms of ADHD and other mental disorders, will impact on both academic adjustment and academic performance during later years of study. However, given the finding that ADHD symptoms of inattention significantly predicted both academic performance and academic adjustment, it is certainly worth future studies exploring relations between academic adjustment during the early years of university and academic performance in later years.

Our findings are consistent with previous research in emphasizing the significance of addressing the mental well-being of undergraduate students, particularly first-years. Beyond poor academic success, university students with ADHD (with and without comorbid psychiatric disorders) may evince unhealthy behaviors (e.g., poor sleeping habits, lack of physical activity, and medication non-adherence; Bolden et al., 2019; Cavanagh et al., 2016). As symptom severity increases, students are more likely to experience impaired short- and long-term quality of life.

This study adds to a growing literature on the relationship between academic performance and common mental health symptoms. The strengths of our study are that we used standardized psychological scales to measure, in a large cohort of students and while controlling for sociodemographic factors and high school academic performance, the effects of ADHD and comorbid psychiatric symptoms on objectively measured academic outcomes over the course of a full university semester. Furthermore, this study adds to existing knowledge on academic adjustment by focusing on first-year South African university students. These findings are valuable and offer guidelines for future interventions targeting vulnerable students transitioning from high school to university.

## Supplemental Material

sj-docx-1-jad-10.1177_10870547241310659 – Supplemental material for Symptoms of ADHD and Other Common Mental Disorders Influence Academic Success in South African Undergraduates Supplemental material, sj-docx-1-jad-10.1177_10870547241310659 for Symptoms of ADHD and Other Common Mental Disorders Influence Academic Success in South African Undergraduates by Nawal Mohamad, Kim-Louise Rousseau, Fatimah Dowlut, Milton Gering and Kevin G. F. Thomas in Journal of Attention Disorders

## Notes

1 *Academic success* is defined as meeting learning objectives, acquiring desirable skills, being satisfied with academic challenges, and persisting in the presence of frustration (York et al., 2015). Hence, it is often measured by the degree to which students perform well on academic tasks—tests, assignments, and examinations—and the extent to which they remain registered for and complete a degree.
