# Supplementary material for: Symptoms of ADHD and Other Common Mental Disorders Influence Academic Success in South African Undergraduates
Source: J Atten Disord. 2025 Jan 17;29(5):363–86. doi: 10.1177/10870547241310659 (PMC11800717; doi:10.1177/10870547241310659)
Supplement: sj-docx-1-jad-10.1177_10870547241310659 – Supplemental material for Symptoms of ADHD and Other Common Mental Disorders Influence Academic Success in South African Undergraduates [file sj-docx-1-jad-10.1177_10870547241310659.docx]

**Supplementary Material**

**Justification for Using Time Point 1 Data**

**as Sole Estimates of Psychiatric Symptomatology**

We conducted correlational analyses describing the magnitude of association between ASRS / BDI-II / BAI / AUDIT total scores at (a) Time Point 1 and Time Point 2, (b) Time Point 2 and Time Point 3, and (c) Time Point 1 and Time Point 3. Table S1 is a correlation matrix presenting the results of those analyses. The correlations ranged between .64 and .90, with all *p*s < .001. These analyses, then, suggest that self-reported symptoms of ADHD, anxiety, depression, and risky alcohol use were relatively stable over time in our sample. Hence, our decision to use the data collected at Time Point 1 for the inferential tests related to the academic performance outcome variable (i.e., to use the largest possible sample size and thus ensure sufficient statistical power to test the hypotheses) was validated.

**Table S1**

*Correlations Between Total Scores on the AAS, ASRS, BDI-II, BAI, and AUDIT Across Time Points*

| Measure | Time Point 1 | Time Point 2 |
| --- | --- | --- |
| ASRS |  |  |
| Time Point 1 |  |  |
| Time Point 2 | .76*** |  |
| Time Point 3 | .71*** | .72*** |
| BDI-II |  |  |
| Time Point 1 |  |  |
| Time Point 2 | .69*** |  |
| Time Point 3 | .64*** | .73*** |
| BAI |  |  |
| Time Point 1 |  |  |
| Time Point 2 | .72*** |  |
| Time Point 3 | .80*** | .70*** |
| AUDIT |  |  |
| Time Point 1 |  |  |
| Time Point 2 | .88*** |  |
| Time Point 3 | .87*** | .90*** |

*Note.* Statistic presented is Pearson’s correlation coefficient, *r*. ASRS = Adult ADHD Self-Report Scale. BDI-II = Beck Depression Inventory-II. BAI = Beck Anxiety Inventory. AUDIT = Alcohol Use Disorder Identification Test.

* *p* < .05. ** *p* < .01. *** *p* < .001.

To further confirm the appropriateness of our decision to use Time Point 1 data as the sole estimate of ADHD, depression, anxiety, and risky alcohol use symptoms (and, in fact, to confirm the appropriateness of our decision to use the ASRS, BDI-II, BAI, and AUDIT, respectively, to measure those constructs), we ran a basic psychometric analysis on each item’s performance as captured at Time Point 1. This analysis included a correlation matrix, Bartlett’s test of sphericity, and a Keyser-Meyer-Olkin analysis to assess the inter-item correlations and multi-collinearity. We also drew scree plots and derived the eigenvalues to identify, within the current sample’s data, the discrete factors comprising each measure. We could then compare those data to the conventional subscales identified by the developers of the instrument. Finally, we assessed internal consistency with Cronbach’s α and omega (Ω) estimates (the latter included because that statistic is considered a stronger measure given the assumptions of internal consistency; Dunn et al., 2014).

Table S2 presents the results of some of these basic psychometric analyses. Cronbach’s α and Ω estimates of internal consistency reliability exceeded .80 for each scale except the AAS. Notably, these estimates are consistent with those reported in similar studies (see, e.g., Atwoli et al., 2011; Makhubela & Debusho, 2016; Kagee et al., 2015; Young & Mayson, 2010).

**Table S2**

*Psychometric Performance of Scales Measuring Self-Reported Academic Adjustment, ADHD, Depression, Anxiety, and Risky Alcohol Use (N = 506)*

|  |  | Internal Consistency | |  |
| --- | --- | --- | --- | --- |
| Measure | Inter-item Correlation Range I | Cronbach’s α | Ω | No. of Identified Factors |
| AAS | (-.47–.63) | .64 | .74 | 3 |
| ASRS | (.30–.59) | .86 | .88 | 3^*^ |
| BDI-II | (.30–.58) | .89 | .90 | 3 |
| BAI | (.30–.71) | .91 | .92 | 3 |
| AUDIT | (.30–.72) | .81 | .85 | 2 |

*Note*. AAS = Academic Adjustment Scale. ASRS = Adult ADHD Self-Report Scale. BDI-II = Beck Depression Inventory-II. BAI = Beck Anxiety Inventory. AUDIT = Alcohol Use Disorder Identification Test. ^*^ The ASRS clusters consist of inattention and a collapsed cluster for symptoms of hyperactivity and impulsivity.

Regarding the number of different factors on which the items within each scale loaded (see the right-most column of Table Sƒ2), these were largely consistent with the factors identified by the developers of each measure. For instance, our analysis of the BDI-II data and of the BAI data identified three distinct factors in each instrument. These findings are consistent with those reported by (a) Makhubela and Debusho (2016) in their study describing BDI-II psychometric properties in a sample of South African university students, and (b) Kagee et al. (2015) in their study describing BAI psychometric properties in a sample of people living with HIV (PWH). Note, however, that our analysis of the BAI data delivered findings inconsistent with other South African research (e.g., Steele & Edwards, 2008): Where we identified three distinct factors, they identified two.

Our analysis of the AUDIT data identified two factors (drinking frequency and risky drinking patterns), a finding consistent with North American research on the instrument (see, e.g., Maisto et al., 2000). However, in a Brazilian sample of university students the AUDIT was found to have three factors (alcohol consumption, dependence of alcohol consumption, adverse consequences of alcohol consumption; Pereira de Araujo Sousa et al., 2020). Further psychometric analyses of this instrument’s data showed that, at Time Point 1, the distribution was skewed right, indicating that most participants generated relatively low scores on the scale. We did not transform those scores because the AUDIT may not be sensitive to detecting hazardous drinking habits among university students: For instance, it might take 18-year-olds longer than a semester to develop such habits (Lindgren et al., 2016).

Our analysis of the ASRS data identified three factors, a finding consistent with Stanton et al. (2018). They classed the factors as inattention, verbal symptoms of hyperactivity-impulsivity, and motor symptoms of hyperactivity-impulsivity. This discrepancy with Kessler et al.’s (2005) proposed two-factor structure may be explained by those authors’ grouping of hyperactivity and impulsivity into one cluster, following DSM-5 diagnostic criteria (American Psychiatric Association, 2013).

Our analysis of the AAS data identified three factors. Consistent with Anderson et al. (2016), these factors could be labelled as academic lifestyle, academic achievement, and academic motivation. Further psychometric analysis of the AAS identified three items with poor inter-item correlations (< .30). We chose to retain those items in our final dataset because (a) internal consistency estimates remained relatively unchanged when they were removed, and (b) we did not want to compromise the scale by shortening it even further from its brief 9-item form.
